# A Temperature Drift Compensation Method for Pulsed Eddy Current Technology

**DOI:** 10.3390/s18061952

**Published:** 2018-06-15

**Authors:** Biting Lei, Pengxing Yi, Yahui Li, Jiayun Xiang

**Affiliations:** School of Mechanical Science & Engineering, Huazhong University of Science & Technology, Wuhan 430074, China; bitinglei@hust.edu.cn (B.L.); liyh20@163.com (Y.L.); m201770406@hust.edu.cn (J.X.)

**Keywords:** pulsed eddy current (PEC), temperature drift, temperature compensation

## Abstract

Pulsed eddy current (PEC) technology is another important non-contact nondestructive testing technology for defect detection. However, the temperature drift of the exciting coil has a considerable influence on the precision of PEC testing. The objective of this study is to investigate the temperature drift effect and reduce its impact. The temperature drift effect is analyzed theoretically and experimentally. The temperature drift effect on the peak-to-peak values of the output signal is investigated, and a temperature compensation method is proposed to reduce the effect of temperature variation. The results show that temperature drift has a negative impact on PEC testing and the temperature compensation method can effectively reduce the effect of temperature drift.

## 1. Introduction

Pulsed eddy current (PEC) technology is another important and effective electromagnetic nondestructive testing technology which possesses many advantages including more extended detection depth, richer information, higher sensitivity and robustness [1]. PEC testing has found application in defect detection in multi-layered structures [2,3,4], stress measurement [5], metal thickness measurement [6], corrosion detection [7,8], and so on. Accuracy and sensitivity are essential for PEC testing. Many factors are likely to affect the accuracy and sensitivity of PEC testing, such as lift-off noise, tilt effects, geometric size of PEC probe, noise, temperature drift effect, etc. However, the effect of temperature drift is ignored by various scholars.

From previous studies, factors that affect the accuracy and sensitivity of PEC testing have attracted the attention of numerous scholars. Yu [9] et al. analyzed the lift-off effect and proposed an approach to reduce the effect of lift-off noise. This study found that the slope of the linear curve of the peak value of the difference signal and the lift-off is tied to the depth and width of the defect. Tian and Sophian [10] proposed an approach using normalization and two reference signals from air measurement and defect-free sample measurement to reduce the lift-off problem with PEC techniques. In addition, Mandache and Lefebvre [11] investigated the lift-off intersection (LOI) which can be utilized to eliminate lift-off noise. Le Bihan [12] studied the effect of the variation in the positioning of a sensor and found that the tilt and the lift-off of the sensor relative to the part under evaluation induce similar straight lines in the normalized impedance plane. In order to improve detection sensitivity of the probe, Arjun [13] optimized the configuration of the probe through finite element modeling. The research carried out by Zhou et al. [14] was concentrated on the optimization of coil parameters to improve the sensitivity of the PEC probe. Moreover, Zhou reduced Gaussian noise of PEC signal effectively using a new de-noising approach based on singular-value decomposition [15].

Some researchers have considered the effect of the working environment temperature on eddy current sensors. Wang and Feng investigated the thermal drift of eddy current sensors and proposed a self-temperature compensation method to reduce thermal drift [16,17]. Zheng, Wu, and Yang analyzed the relationship between the eddy current sensor output and temperature variation, and compensated for the temperature drift of the sensor using a temperature-voltage curve equation [18]. However, few researchers have considered the effect of the temperature of the exciting coil on PEC testing.

In this paper, we mainly discuss the effect of temperature drift caused by the change in temperature of the exciting coil in PEC testing, and how to reduce or eliminate the effects of temperature. The relationship between the peak-to-peak values of the output signal and the temperature variation is analyzed and a temperature compensation method is proposed to reduce or even eliminate the influence of temperature drift. The rest of the paper is structured as follows. Section 2 analyzes temperature drift by the theoretical method. Section 3 presents the experimental set-up and sample. Section 4 proposes a temperature compensation method. Section 5 discusses and analyzes the result of compensation. Section 6 presents the conclusions.

## 2. Temperature Drift Effect in Pulsed Eddy Current Testing

The basic principle of PEC testing is electromagnetic induction, which occurs when the exciting coil carrying varying current is positioned in proximity to the testing sample. The eddy current will be produced in the surface of the sample. The eddy current in the sample will change with variations in the electrical conductivity or magnetic permeability of the test specimen, or the presence of any flaws. In addition, the entire magnetic field will change with a change in the eddy current. Through observing changes in the magnetic field using a pickup coil or a magnetic sensor (e.g., Hall sensor, Giant magnetoresistance (GMR)) [19], electrical conductivity or magnetic permeability of the test specimen, or flaws, can be detected.

**Figure 1:**
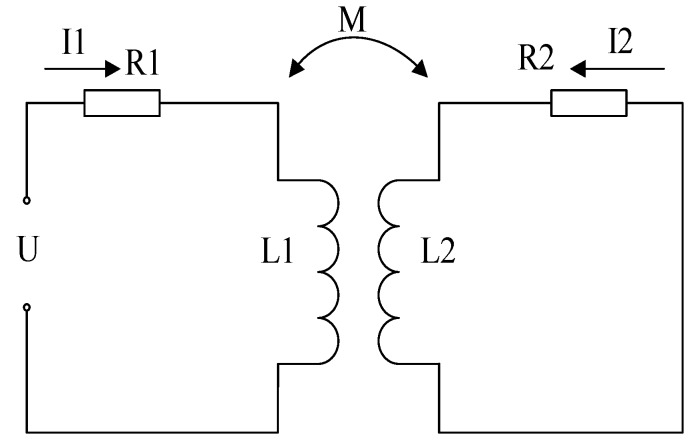
The equivalent circuit of the PEC testing system.

The PEC testing system is equivalent to Figure 1 [17,20], where *R*_1_, *L*_1_ is the resistance and inductance of the exciting coil, respectively. *R*_2_, *L*_2_ is the equivalent resistance and inductance caused by eddy current in the sample. Based on the Kirchhoff’s voltage laws, we will get:

(1) $$\left\{ \begin{array}{l} I_{1}R_{1}+I_{1}j2\pi fL_{1}-I_{2}j2\pi fM=U\\ -I_{1}j2\pi fM+I_{2}R_{2}+I_{2}j2\pi fL_{2}=0 \end{array}\right.$$

where *U* is the excitation voltage; *f* is the excitation frequency of coil; *M* is the mutual inductance between the coil and the sample. Based on Formula (1), the equivalent resistance and inductance of the PEC system can be obtained.

(2) $$R=R_{1}+\frac{{\left(2\pi f\right)}^{2}M^{2}}{R_{2}^{2}+{\left(2\pi fL_{2}\right)}^{2}}R_{2}$$

(3) $$L=L_{1}-\frac{{\left(2\pi f\right)}^{2}M^{2}}{R_{2}^{2}+{\left(2\pi fL_{2}\right)}^{2}}L_{2}$$

For the equivalent resistance and inductance in Formulae (2) and (3), if the lift-off distance remains unchanged, the effective impedance affected by temperature can be expressed as:

(4) $$\begin{array}{l} Z_{eff}=R(T)+j\omega L(T)\\ =R_{1}(T)+R_{2}(T)+j\omega (L_{1}(T)+L_{2}(T)) \end{array}$$

Since the material of the exciting coil is copper, the temperature coefficient of *R*_1_ is about 3900 ppm/°C and the temperature coefficient of *L*_1_ is about 10–100 ppm/°C. Inductance *L* is only affected by the geometry of the cylinder inductor and the number of turns of enamel-insulated wire wound. The geometric size changes fractionally with the temperature. However, a low thermal expansion frame will contribute significantly to the stability of *L*. Experimental data show that the value of the inductance *L* changes only 0.27% for the temperature change of 150 °C, so the change of excitation coil inductance L affected by temperature can be ignored. 

The material of the sample is aluminum. The temperature coefficient of *L*_2_ is around several hundred ppm/°C, but that of *R*_2_ is usually more than 2000 ppm/°C. Thus, the effect of temperature on *L*_2_ is smaller than on *R*_2_ [17]. However, temperature variation in the PEC system is mainly caused by changes in the exciting coil temperature. Therefore, the temperature variation of the sample can be ignored.

Based on the analysis, the change in effective impedance for the effect of temperature depends on the change in *R*_1_. In Formula (5), *R*_0_ is the resistance at 0 °C, a denotes the temperature coefficient, $a=4.3\times 10^{-3}$(1/°C).

(5) $$R_{1}=R_{0}(1+\alpha T)$$

The resistance *R*_1_ will increase with the increasing temperature of the exciting coil. Then, the effective impedance, *Z_eff_*, will also increase, which will hamper the original magnetic field excited by the exciting coil, and then the output of the Hall sensor will be impeded. Therefore, the output of the signal will decline as the temperature of the exciting coil in the PEC probe increases. 

## 3. Experimental Set-Up and Sample

**Figure 2:**
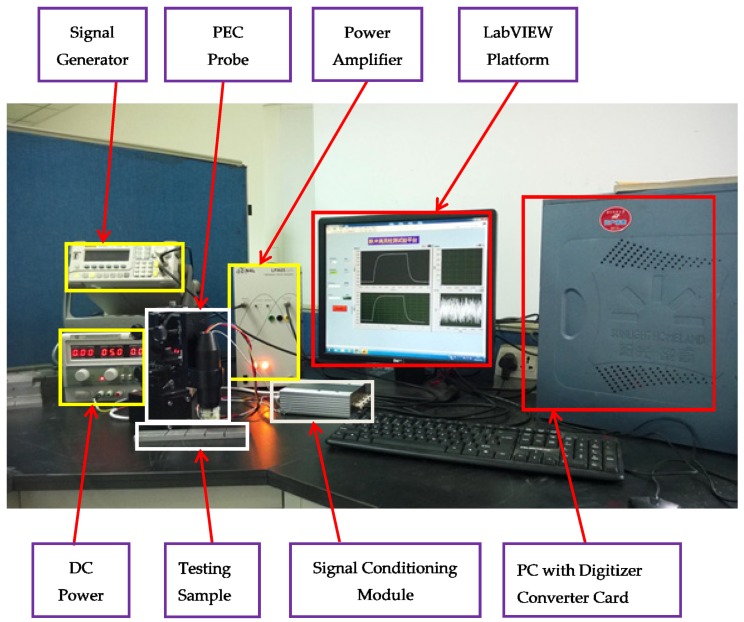
A standard PEC testing system.

As shown in Figure 2, the PEC experimental testing device consisted of a function signal generator, power amplifier, DC power, a signal conditioning module, digitizer converter card, testing sample, the probe and a computer system with LabView software.

**Figure 3:**
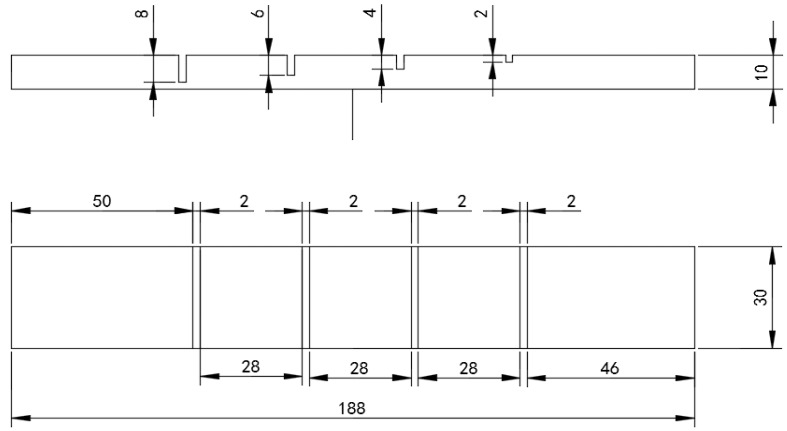
The geometric parameters of the PEC testing sample.

As shown in Figure 3, a 7075 aluminum alloy plate of 10 mm thickness, 188 mm length and 30 mm width was used for the experiment. Four cracks were fabricated in the aluminum plate surface of width 2 mm and depths of 2 mm, 4 mm, 6 mm, and 8 mm, respectively. A probe with a cylindrical coil was placed directly on the specimen to detect defects. The outer diameter, inner diameter, and height of the cylindrical coil was 32 mm, 15 mm, and 20 mm respectively. The coil was made of copper enameled wire with a diameter of 1 mm. The number of turns of the coil was 75. The excitation current in the cylindrical coil was a rectangular pulse signal (100 Hz and 400 mV). The signal was excited by a functional signal generator and amplified 10 times by the power amplifier. A Hall sensor (95 A) was placed directly below the symmetry axis of the coil and was utilized to obtain the change in electromagnetic fields from the sample and the excitation coil. The Hall sensor output a voltage signal containing defect information. The output voltage signal was relatively small and had to be amplified, so the amplifier gain was fixed at 100 times to meet the measurement range of the digitizer card. The digitizer converter card was utilized to convert the analog signal to a digital signal. The sampling frequency of the digitizer converter card was 100 kHz with 16-bit resolution. LabView software was utilized to store the signal on the PC. 

In the experiment, the aluminum plate with defects of depth 0 mm, 2 mm, 4 mm, 6 mm, and 8 mm, was measured under different temperatures. The temperatures varied from 24 °C to 57 °C. The variation step of temperature was 3 °C. The experimental data are listed in Table 1.

## 4. Temperature Compensation Method

Based on the above discussion, we can see that the output voltage of PEC testing changes as the temperature of the exciting coil changes. In this paper, a compensation method based on the binary regression method is proposed. When the aluminum plate surface has no defect, the output is a function of the temperature of the exciting coil. The relationship can be characterized by Equation (6). When the aluminum plate surface has a defect, the output is related to the temperature of the exciting coil and the size of the defect. The relationship can be characterized by Equation (7).

(6) $$u_{f}=f(t)$$

(7) $$u_{h}=g(t,h)$$

$u_{f}$ denotes the peak-to-peak values of the output signal with no defect, as the reference signal, $u_{h}$ denotes the peak-to-peak values of the output signal with a defect, *t* denotes the temperature, *h* denotes the depth of the defect.

Equations (6) and (7) yield,

(8) $$t=f^{-1}(u_{f})$$

(9) $$h=p(u_{h},t)=p(u_{h},f^{-1}(u_{f}))=q(u_{h},u_{f})$$

The relationship between the defect depth, temperature and output voltage can be transformed into the relationship between the defect depth, reference values, and output voltage. However, temperature compensation must be performed in order to improve measurement accuracy. We use a two-dimensional regression method to establish the corresponding relationship between the defect depth, reference values, and output voltage. The coefficient of the regression equation was developed using the least squares fitting method.

The regression equation is below:

(10) $$h=a_{0}+a_{1}u_{f}+a_{2}u_{h}+a_{3}{u_{f}}^{2}+a_{4}u_{f}u_{h}+a_{5}{u_{h}}^{2}$$

In order to obtain the coefficient $a_{i},\left(i=1,2\cdots 5\right)$. Seek $a_{i}$ to make sure that:

(11) $$R={\left(h-h_{0}\right)}^{2}=\min$$

So,

(12) $$R=\sum_{k=1}^{m\times n}{\left(a_{0}+a_{1}u_{f}+a_{2}u_{hk}+a_{3}{u_{fk}}^{2}+a_{4}u_{fk}u_{hk}+a_{5}{u_{hk}}^{2}-h_{0k}\right)}^{2}$$

$h_{0k}$ denotes the actual defect depth; k=1, 2⋯m×n.

This is a multivariate function referred to the coefficient, $a_{i}$. According to the essential condition of finding the extreme value of a multivariate function, let each partial derivative be zero and obtain the value of the coefficient.

That is:

(13) $$\frac{\partial R}{\partial a_{i}}=0,\left(i=1,2\cdot \cdot \cdot 5\right)$$

Substituting Equation (7) into Equation (8), yield,

(14) $$\left[\begin{array}{cccccc} m\times n & \sum_{1}^{m\times n}u_{fk} & \sum_{1}^{m\times n}u_{hk} & \sum_{1}^{m\times n}{u_{fk}}^{2} & \sum_{1}^{m\times n}u_{fk}u_{hk} & \sum_{1}^{m\times n}{u_{hk}}^{2}\\ \sum_{1}^{m\times n}u_{fk} & \sum_{1}^{m\times n}{u_{fk}}^{2} & \sum_{1}^{m\times n}u_{fk}u_{hk} & \sum_{1}^{m\times n}{u_{fk}}^{3} & \sum_{1}^{m\times n}{u_{fk}}^{2}u_{hk} & \sum_{1}^{m\times n}u_{fk}{u_{hk}}^{2}\\ \sum_{1}^{m\times n}u_{hk} & \sum_{1}^{m\times n}u_{fk}u_{hk} & \sum_{1}^{m\times n}{u_{hk}}^{2} & \sum_{1}^{m\times n}{u_{fk}}^{2}u_{hk} & \sum_{1}^{m\times n}u_{fk}{u_{hk}}^{2} & \sum_{1}^{m\times n}{u_{hk}}^{3}\\ \sum_{1}^{m\times n}{u_{fk}}^{2} & \sum_{1}^{m\times n}{u_{fk}}^{3} & \sum_{1}^{m\times n}{u_{fk}}^{2}u_{hk} & \sum_{1}^{m\times n}{u_{fk}}^{4} & \sum_{1}^{m\times n}{u_{fk}}^{3}u_{hk} & \sum_{1}^{m\times n}{u_{fk}}^{2}{u_{hk}}^{2}\\ \sum_{1}^{m\times n}u_{fk}u_{hk} & \sum_{1}^{m\times n}{u_{fk}}^{2}u_{hk} & \sum_{1}^{m\times n}u_{fk}{u_{hk}}^{2} & \sum_{1}^{m\times n}{u_{fk}}^{3}u_{hk} & \sum_{1}^{m\times n}{u_{fk}}^{2}{u_{hk}}^{2} & \sum_{1}^{m\times n}u_{fk}{u_{hk}}^{3}\\ \sum_{1}^{m\times n}{u_{hk}}^{2} & \sum_{1}^{m\times n}u_{fk}{u_{hk}}^{2} & \sum_{1}^{m\times n}{u_{hk}}^{3} & \sum_{1}^{m\times n}{u_{fk}}^{2}{u_{hk}}^{2} & \sum_{1}^{m\times n}u_{fk}{u_{hk}}^{3} & \sum_{1}^{m\times n}{u_{hk}}^{4} \end{array}\right]\left[\begin{array}{c} a_{0}\\ a_{1}\\ a_{2}\\ a_{3}\\ a_{4}\\ a_{5} \end{array}\right]=\left[\begin{array}{c} \sum_{1}^{m\times n}h_{0k}\\ \sum_{1}^{m\times n}u_{fk}h_{0k}\\ \sum_{1}^{m\times n}u_{hk}h_{0k}\\ \sum_{1}^{m\times n}u_{fk}{}^{2}h_{0k}\\ \sum_{1}^{m\times n}u_{fk}u_{hk}h_{0k}\\ \sum_{1}^{m\times n}u_{hk}{}^{2}h_{0k} \end{array}\right]$$

From Equation (14), $a_{i},\left(i=1,\;2\cdots 5\right)$ can be solved quickly. Thus, the regression equation is worked out. Thus, the defect depth after compensation is obtained. In the actual measurement, if the reference signal and the output signal with a defect at the same temperature are obtained, the defect depth after compensation can be obtained according to Equation (10).

## 5. Result and Discussion

**Figure 4:**
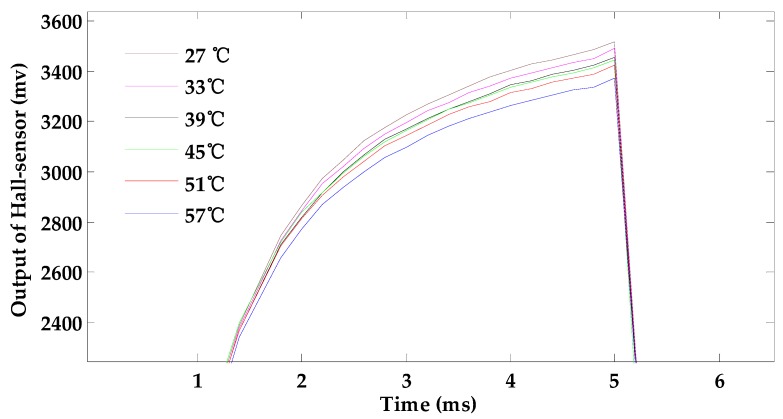
The effect of temperature rises in the exciting coil on the testing signal.

**Figure 5:**
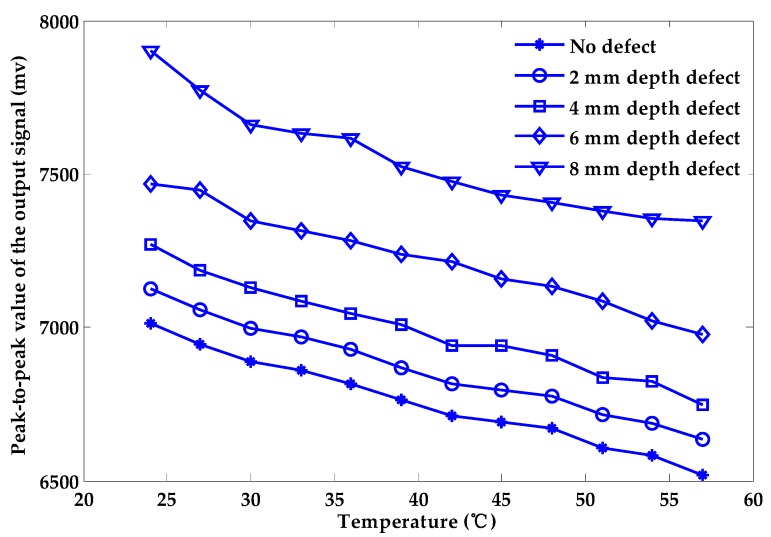
The relationship between the peak-to-peak value of the output signal and the temperature.

Figure 4 shows the influence of temperature variation on the output signal of the Hall sensor under the 2 mm-deep defect. With the increasing temperature, the output of the Hall sensor decreases. The trend is in line with the theoretical result. Measuring the depth of the defect in the sample shown in Figure 3, the changes in peak-to-peak values of the output signal of the Hall sensor under different temperatures are given in Figure 5. The peak-to-peak values of the output signal of the Hall sensor decrease linearly approximately with increasing temperature. Therefore, temperature variation has a negative effect on the quantitative characterization process of surface depth defect.

**Figure 6:**
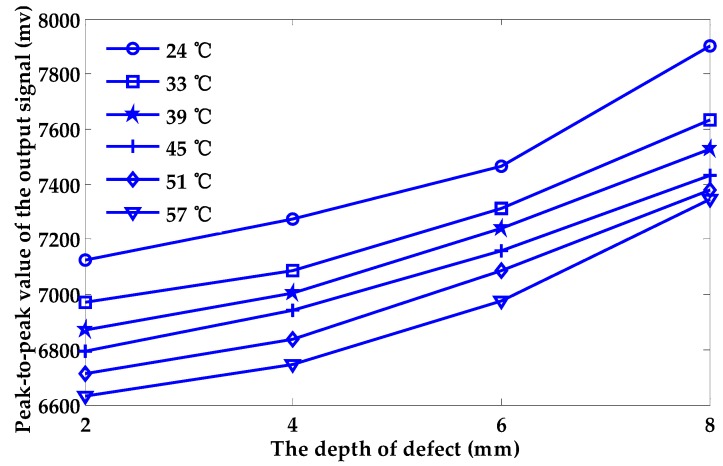
The relationship between the peak-to-peak value of the output signal and the depth of the defect at different temperatures.

**Figure 7:**
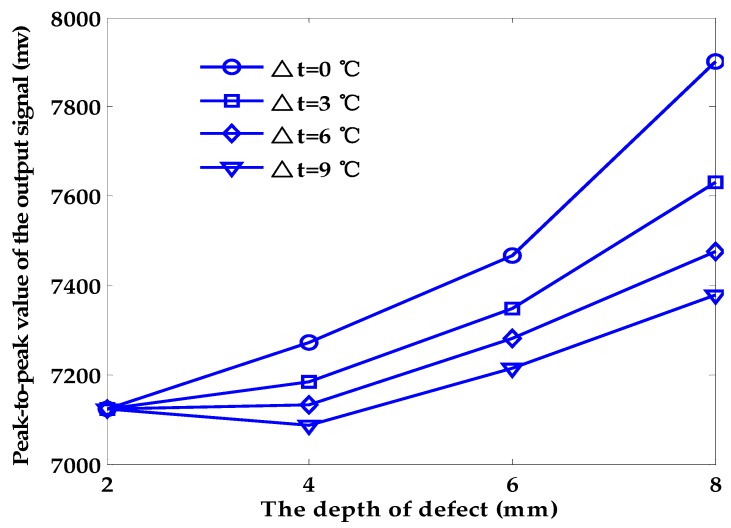
Temperature drift effect on the quantitative characterization of flaws in depth.

Figure 6 shows the relationship between the peak-to-peak values of the output signal and the depth of the defect under different temperatures of exciting coil. At the same temperature, the peak-to-peak values and the depth of defects are roughly linear. However, when the variation interval of temperature is 3 °C, 6 °C and 9 °C, respectively, the relationship between the peak-to-peak values of the output signal and the depth of the defect cannot be described by fixed equations, as shown in Figure 7. We can conclude that the effect of the temperature rise in the exciting coil will have a huge negative impact on the PEC testing process and greatly lower the accuracy of the quantitative characterization process. Considerable errors will arise when quantifying the depth of a surface defect without considering any temperature change in the exciting coil.

From Figure 6, the relationship between the peak-to-peak value of the output signal and the depth of the defect can be described by a function of the first degree when the temperature remains unchanged. In this paper, the defect depth with no compensation ($D_{nc}$) can be calculated using Equation (15), which is the fitting equation when the temperature of the coil is 24 °C and the temperature variation is not considered. Based on the temperature compensation method stated in Section 4, the depth of defects after compensation ($D_{c}$) is listed in Table 2.

(15) $$D_{nc}=0.00791*u_{h}-53.818$$

Reference [21] compensates for the variation of the temperature based on 3D surface fitting and two-dimensional curved-surface interpolation. The consequence of compensation using 3D surface fitting method is listed in Table 2, compared with the result of using the binary regression method stated in this paper.

From the data in Table 2, we can conclude that the less the temperature of the exciting coil increases, the less the influence of temperature drift on the quantitative characterization of the depth of surface defect, and with a large deviation of the initial temperature of the exciting coil some of the results may even be incorrect.

**Figure 8:**
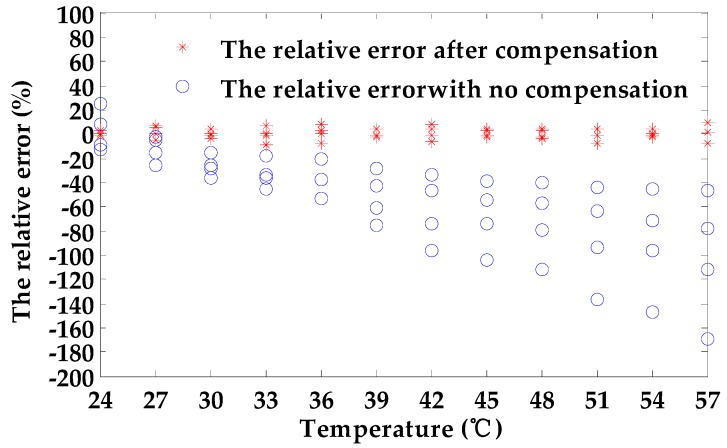
The distribution of the relative error with and without compensation.

**Figure 9:**
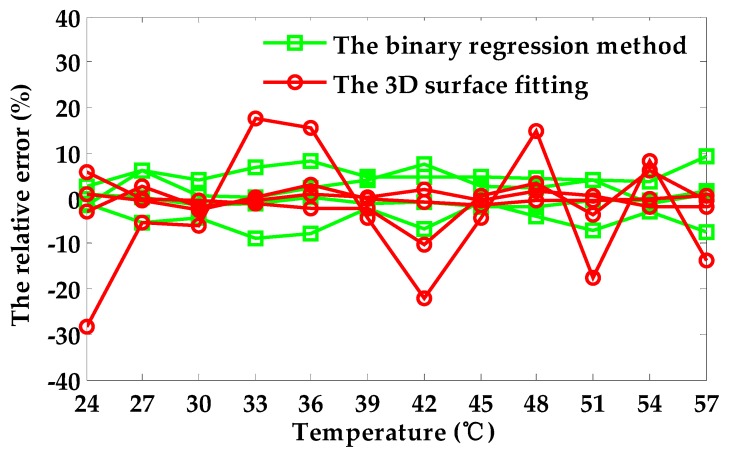
The comparison of the compensation error using two different methods.

Figure 8 displays the distribution of the relative error. When the depth of defects is obtained without temperature compensation, the maximal relative measurement error is 169.08%. Therefore, the temperature drift of the coil has a serious impact on the measurement of defect depth. However, the maximal relative error obtained by the temperature compensation method based on the binary regression method is 9.13%, which is considerably less than the relative error without the temperature compensation method. Figure 9 displays the comparison of the compensation error using the binary regression method and 3D surface fitting. The maximal relative error using 3D surface fitting is 28.2%, which is greater than the maximal relative error using the binary regression method. Additionally, the distribution of the relative error using the binary regression method is more uniform than using 3D surface fitting. The results indicate that the temperature compensation method is effective in reducing or even eliminating the effects of temperature drift.

## 6. Conclusions

In this paper, we have mainly discussed the effect of exciting coil temperature variation on PEC testing and proposed a compensation method which aims to reduce or even eliminate the effect of temperature drift. Specifically, the detailed conclusions are summarized as follows.

(1) According to theoretical analysis, the output of the signal decreases when the temperature increases for the change in the impedance of the exciting coil and the magnetic field distribution. The temperature variation of the exciting coil has a considerable influence on the accuracy of PEC testing.
(2) According to experimental analysis, the peak-to-peak values of the output signal decrease as the temperature of the exciting coil in the PEC probe rises. In addition, the temperature variation of the exciting coil has a depressing influence on the quantitative characterization of surface flaw depth.
(3) A temperature compensation method based on the binary regression method was proposed to reduce the temperature drift effect. We verified the effectiveness of the proposed method through the comparative analysis. Our work indicates that the method is capable of compensating for the temperature variation.

However, much more work is still needed. We could analyze the influence caused by the temperature variation of PEC probe using the finite element analysis (FEA) method. The relationship between other features and temperature and compensation method could be investigated. Additionally, we could verify the applicability of the proposed method to other features. Finally, the practical application and usability of this temperature compensation method of PEC testing have yet to be improved.

## Figures and Tables

**Table 1:** The peak-to-peak values of the output signals under different temperatures.

Temperature (°C)	The Actual Depth of Defects (mm)				
	0	2	4	6	8
24	7013.13	7123.57	7271.38	7467.02	7901.54
27	6944.18	7056.57	7184.1	7447.01	7773.56
30	6888.68	7010.11	7130.74	7347.5	7660.92
33	6859.58	6970.21	7085.26	7312.56	7631.63
36	6815.39	6926.95	7043.06	7282.43	7615.1
39	6762.32	6882.68	6948.44	7238.39	7525.41
42	6711.36	6851.61	6941.67	7213.61	7475.45
45	6690.25	6809.01	6940.5	7157.67	7430.93
48	6669.88	6804.93	6909.31	7133.42	7408.1
51	6607.28	6742.6	6837.68	7084.27	7377.75
54	6580.87	6701.5	6763.17	7021.54	7355.4
57	6516.55	6633.23	6748.21	6975.36	7346.26

**Table 2:** Comparison of the relative error.

The Actual Defect Depth *D_a_* (mm)	Defect Depth with No Compensation *D_nc_* (mm)	Defect Depth Compensated Using the Method in This Paper *D_c_* (mm)	Defect Depth Compensated Using the Method in Reference [21] *D_c_′* (mm)	The Relative Error of *D_a_* and *D_nc_*(%)	The Relative Error of *D_a_* and *D_c_*(%)	The Relative Error of *D_a_* and *D_c_′* (%)
2	2.4946	2.050891	1.4360	24.73	2.5445	−28.2
2	1.9650	2.122814	1.8894	−1.75	6.1407	−5.53
2	1.4880	2.081665	1.8777	−25.60	4.0833	−6.115
2	1.2823	2.135864	2.3535	−35.89	6.7932	17.675
2	0.9403	2.161226	2.3111	−52.98	8.0613	15.555
2	0.4793	2.095173	1.9126	−76.03	4.7587	−4.37
2	0.0755	2.094052	1.5574	−96.22	4.7026	−22.13
2	−0.0913	2.092481	1.9103	−104.56	4.6241	−4.485
2	−0.2520	2.090351	2.2941	−112.60	4.5176	14.705
2	−0.7441	2.080029	1.6482	−137.21	4.0014	−17.59
2	−0.9510	2.073965	2.1616	−147.55	3.6982	8.08
2	−1.3816	2.182746	1.7235	−169.08	9.1373	−13.825
4	3.6631	3.945851	4.2341	−8.42	−1.354	5.8525
4	2.9731	3.777778	3.9897	−25.67	−5.556	−0.2575
4	2.5513	3.832751	3.9733	−36.22	−4.181	−0.6675
4	2.1918	3.6472	3.9505	−45.21	−8.82	−1.2375
4	1.8582	3.685564	3.9094	−53.55	−7.861	−2.265
4	1.5743	3.902831	3.9163	−60.64	−2.429	−2.0925
4	1.0567	3.730847	3.5859	−73.58	−6.729	−10.3525
4	1.0474	3.969954	4.0167	−73.81	−0.751	0.4175
4	0.8009	3.838789	4.1333	−79.98	−4.03	3.3325
4	0.2346	3.716813	3.8482	−94.13	−7.08	−3.795
4	0.1387	3.881465	4.2477	−96.53	−2.963	6.1925
4	−0.4726	3.695957	3.9774	−111.82	−7.601	−0.565
6	5.2096	5.910945	5.8148	−13.17	−1.484	−3.087
6	5.0515	6.359941	6.1588	−15.81	5.999	2.647
6	4.2648	6.039267	5.8774	−28.92	0.6545	−2.043
6	3.9910	6.00673	5.9752	−33.48	0.1122	−0.413
6	3.7504	6.140391	6.0619	−37.49	2.3398	1.032
6	3.4023	6.229734	6.0198	−43.30	3.8289	0.33
6	3.2064	6.446525	6.1160	−46.56	7.4421	1.933
6	2.7642	6.16644	5.9744	−53.93	2.774	−0.427
6	2.5725	6.133711	6.0883	−57.13	2.2285	1.472
6	2.1840	6.238594	6.0278	−63.60	3.9766	0.463
6	1.6881	5.922512	5.8872	−71.87	−1.291	−1.88
6	1.3230	6.057296	5.8794	−77.95	0.9549	−2.01
8	8.6446	8.062432	8.0745	8.06	0.7804	0.931
8	7.6329	8.011431	7.9598	−4.59	0.1429	−0.503
8	6.7425	7.886739	7.8002	−15.72	−1.416	−2.498
8	6.5109	7.903787	8.0025	−18.61	−1.203	0.0312
8	6.3802	8.014011	8.2355	−20.25	0.1751	2.944
8	5.6712	7.915649	7.9895	−29.11	−1.054	−0.131
8	5.2763	7.931744	7.9348	−34.05	−0.853	−0.815
8	4.9243	7.847104	7.8852	−38.45	−1.911	−1.435
8	4.7439	7.839165	7.9524	−40.70	−2.01	−0.595
8	4.5040	7.958886	7.9527	−43.70	−0.514	−0.591
8	4.3273	7.96967	7.9805	−45.91	−0.379	−0.244
8	4.2550	8.120239	8.0530	−46.81	1.503	0.6625

## References

[1] He Y., Luo F., Pan M., Hu X., Gao J., Liu B. (2010). Defect classification based on rectangular pulsed eddy current sensor in different directions. Sens. Actuators A Phys..

[2] He Y., Luo F., Hu X., Liu B., Gao J. (2009). Defect identification and evaluation based on three-dimensional magnetic field measurement of pulsed eddy current. Insight Non-Destr. Test. Cond. Monit..

[3] He Y., Luo F., Pan M., Weng F., Hu X., Gao J., Liu B. (2010). Pulsed eddy current technique for defect detection in aircraft riveted structures. NDT E Int..

[4] Yu Y., Guan J. (2013). Investigation of signal features of pulsed eddy current testing technique by experiments. Insight.

[5] Morozov M., Yun T.G., Withers P.J. (2010). The pulsed eddy current response to applied loading of various aluminium alloys. NDT E Int..

[6] Yang H.C., Tai C.C. (2002). Pulsed eddy-current measurement of a conducting coating on a magnetic metal plate. Meas. Sci. Technol..

[7] Moulder J.C., Bieber J.A., Crane R.L., Achenbach J.D., Shah S.P., Matikas T.E., KhuriYakub P.T., Gilmore R.S. (1998). Pulsed eddy-current measurements of corrosion and cracking in aging aircraft. Nondestructive Characterization of Materials in Aging Systems.

[8] Krause T.W., Harlley D., Babbar V.K., Wannamaker K., Thompson D.O., Chimenti D.E. (2010). Pulsed Eddy Current Thickness Measurement Of Selective Phase Corrosion on Nickel Aluminum Bronze Valves. AIP Conf. Proc..

[9] Yu Y., Yan Y., Wang F., Tian G., Zhang D. (2014). An approach to reduce lift-off noise in pulsed eddy current nondestructive technology. NDT E Int..

[10] Tian G.Y., Sophian A. (2005). Reduction of lift-off effects for pulsed eddy current NDT. NDT E Int..

[11] Mandache C., Lefebvre J.H.V. (2006). Transient and harmonic eddy currents: Lift-off point of intersection. NDT E Int..

[12] Le Bihan Y. (2002). Lift-off and tilt effects on eddy current sensor measurements: A 3-D finite element study. Eur. Phys. J. Appl. Phys..

[13] Arjun V., Sasi B., Rao B.P.C., Mukhopadhyay C.K., Jayakumar T. (2015). Optimisation of pulsed eddy current probe for detection of sub-surface defects in stainless steel plates. Sens. Actuators A Phys..

[14] Zhou D., Tian G.Y., Li Y. (2010). Simulation based on optimisation of pulsed eddy current probe design. Nondestruct. Test. Eval..

[15] Zhu H., Wang C., Chen H., Wang J. (2015). Pulsed eddy current signal denoising based on singular value decomposition. J. Shanghai Jiaotong Univ. (Sci.).

[16] Wang H., Ju B., Li W., Feng Z. (2014). Ultrastable eddy current displacement sensor working in harsh temperature environments with comprehensive self-temperature compensation. Sens. Actuators A Phys..

[17] Wang H., Feng Z. (2013). Ultrastable and highly sensitive eddy current displacement sensor using self-temperature compensation. Sens. Actuators A Phys..

[18] Zheng Y., Wu J., Yu Y. Temperature compensation of eddy current sensor based on temperature-voltage model. Proceedings of the 12th World Congress on Intelligent Control and Automation (WCICA).

[19] Angani C.S., Park D.G., Kim C.G., Leela P., Kollu P., Cheong Y.M. (2010). The Pulsed Eddy Current Differential Probe to Detect a Thickness Variation in an Insulated Stainless Steel. J. Nondestruct. Eval..

[20] Tian G.Y., Zhao Z.X., Baines R.W. (1998). Baines. The research of inhomogeneity in eddy current sensors. Sens. Actuators A Phys..

[21] Li Y. (2016). A Method to Detect and Quantitate Flaw with Pulsed Eddy Current Technology Considering Temperature Drift Effect. Master’s Thesis.

